# (Pro)renin receptor promotes colorectal cancer progression through inhibiting the NEDD4L-mediated Wnt3 ubiquitination and modulating gut microbiota

**DOI:** 10.1186/s12964-022-01015-x

**Published:** 2023-01-03

**Authors:** Juan Wang, Yuwei Ding, Dan Li, Ning Zhu, Akira Nishiyama, Ying Yuan

**Affiliations:** 1 Department of Medical Oncology (Key Laboratory of Cancer Prevention and Intervention, China National Ministry of Education, Key Laboratory of Molecular Biology in Medical Sciences of Zhejiang Province), The Second Affiliated Hospital, Zhejiang University School of Medicine, Hangzhou, 310009 Zhejiang China; 2 Zhejiang Provincial Clinical Research Center for Cancer, Hangzhou, 310009 Zhejiang China; 3 Cancer Center of Zhejiang University, Hangzhou, 310058 Zhejiang China; 4 Department of Pharmacology, Faculty of Medicine, Kagawa University, Takamatsu, Kagawa 761-0793 Japan

**Keywords:** Colorectal cancer, Progression, (Pro)renin receptor, Wnt3 ubiquitination, NEDD4L, Gut microbiota

## Abstract

**Background:**

We previously found that (pro)renin receptor ((P)RR) augments Wnt3 protein without affecting *Wnt3* gene transcription in colorectal cancer (CRC) cells, thus contributes to CRC initiation. The present study aims to investigate whether (P)RR further promotes CRC progression following oncogenesis and the related mechanisms. Notably, we deeply elaborate how (P)RR affects Wnt3 protein level and the key enzyme that mediates this process.

**Methods:**

Immunohistochemistry, western blotting and immunofluorescence were performed to detect protein expression status. A kind of gastrointestinal epithelium-specific *ATP6AP2* ((P)RR encoding gene) knock-in mice were generated using Crispr/Cas9 system.

**Results:**

We found that increased (P)RR expression in primary CRC lesions is positively associated with higher Wnt3 protein level and disease progression. Progressive CRC presents less colocalization of Wnt3 and an E3 ubiquitin ligase NEDD4L in primary lesions than non-progressive CRC. In colon cancer cells, (P)RR dramatically inhibits the NEDD4L-mediated Wnt3 protein ubiquitination. *ATP6AP2* knock-in mice show more diminished Wnt3-NEDD4L colocalization in their gut epithelium in comparison to wildtype mice. They also have abnormal gut bacterial flora distribution. Especially, Lachnospiraceae_NK4A136 and Bacteroides genus, which are generally protective against CRC, are suppressed in guts of *ATP6AP2* knock-in mice.

**Conclusions:**

Collectively, (P)RR promotes CRC progression through inhibiting the NEDD4L-mediated Wnt3 ubiquitination and modulating gut microbiota.

Video Abstract

**Supplementary Information:**

The online version contains supplementary material available at 10.1186/s12964-022-01015-x.

## Background

Colorectal cancer (CRC) is a kind of malignancy with the fourth highest morbidity and second highest mortality worldwide [1]. Only about 39% of patients with CRC are diagnosed in the early stage, while most are already in the middle or advanced stage when the disease is found. Moreover, the respective 5 year survival rates of CRC patients with local and distant metastasis are only 53% and 11% [2]. Liver is the most common metastatic site of CRC, and liver metastasis is also the leading cause of CRC-related death. About 30%-50% of CRC patients are found with liver metastasis at the time of diagnosis or in a period after radical resection of the primary tumor, and 80%-90% of them lose the opportunity of further radical surgery to completely remove the metastatic lesions.

Excessive activation of Wnt/β-catenin signaling pathway (hereinafter also referred to as "Wnt signaling pathway") leads to aberrant cell proliferation, cell apoptosis suppression, epithelial-mesenchymal transformation (EMT), immune escape and other carcinogenetic behaviors of cells, thus results in the occurrence, progression and metastasis of various cancers including CRC [3–6]. Activation of Wnt signaling pathway starts from the binding of Wnt ligands to Wnt receptor complex, which is mainly composed of LRP6, Frizzeled and (pro)renin receptor ((P)RR) [7]. Interestingly, we previously revealed that (P)RR not only acts as a component of Wnt receptor complex, but also associates with the up-regulation of Wnt3 protein in cytoplasm of CRC cells without affecting *Wnt3* gene transcription [8]. Furthermore, (P)RR promotes the occurrence of CRC through enhancing Wnt signaling activity, even if constitutive pathway component mutations exist [8]. However, it is still unclear whether (P)RR further contributes to CRC progression and by which mechanisms (P)RR could influence Wnt3 protein level. Based on above facts, we speculate that (P)RR may inhibit the degradation of Wnt3 protein at post-transcriptional level, and this could partially contribute to CRC progression.

Protein is mainly degraded through proteasome and/or lysosome pathways, notebly, the critical precondition for activating either pathway is protein ubiquitination. Ubiquitination, namely the binding of target protein and ubiquitin (Ub), is regarded as a process of labeling the target protein with Ub. The ubiquitinated protein will then be sent for degradation by proteasome and/or lysosome [9, 10]. E3 ubiquitin ligases (E3s) is a goup of key enzymes that not only mediate ubiquitination but also decide the specific target protien. By direct binding with both target protein and Ub, E3s induce the formation of target protein-E3-Ub complex, thus mediate the ubiquitination and subsequent degradation of target protein [11]. Ding et al. [12] previously revealed that NEDD4L (neural precursor cell expressed developmentally downregulated gene 4-like), an HECT domain-containing E3 ubiquitin ligase, negatively regulates Wnt signaling activity in human CRC cells and acts as a tumor suppressor. However, this effect of NEDD4L could be reversed by adding exogenous active Wnts into cell culture medium [12]. These hints suggest the possibility that NEDD4L mediates the ubiquitination and degradation of endogenous Wnts. In addition, Quadri et al. [13] demonstrated that (P)RR attenuates enzymatic activity of NEDD4-2, the homologous protein of human NEDD4L protein in mice, by mediating NEDD4-2 phospharylation in mouse renal inner medullary collecting duct cells. According to these indirect evidence, this study aims to explicit whether (P)RR inhibits Wnt3 ubiquitination, and if so, whether NEDD4L plays a role in this process.

To fully understand the mechanisms by which (P)RR affects CRC from different aspects, we also investigated the influence of (P)RR on gut microbiota that is demonstrated to be closely relevant to CRC [14, 15]. Generally, gut microbiota influences CRC progression through their metabolic products, physiological activities as attachment, invasion, and translocation, as well as bacteria-host immune cell interactions [15, 16]. Therefore, identification of key factors that cause pathogenic microbial dysbiosis and critical gut microbiota modulator should be important to CRC prevention and therapy. Hence, we also explored whether (P)RR could exert any influence on gut microbiota that affecting CRC progression.

In the present study, we reveal that (P)RR promotes CRC progression through inhibiting the NEDD4L-mediated Wnt3 ubiquitination and modulating gut microbiota. Especially, Lachnospiraceae_NK4A136 and Bacteroides genus, which are generally protective against CRC, could be suppressed by (P)RR. This study provides novel understanding of the roles of (P)RR in CRC progression, which might be further translated into important therapeutic strategy of CRC in the future.

## Materials and methods

### Patients’ tissue samples

This study was conducted in accordance with the Declaration of Helsinki. All procedures were approved by the Ethics Committee of The Second Affiliated Hospital of Zhejiang University School of Medicine (approval number: 2020246). Colon tissue samples were collected from patients pathologically diagnosed with CRC and received surgical resection in The Second Affiliated Hospital, Zhejiang University School of Medicine.

### Immunohistochemistry (IHC), western blotting, immunoprecipitation (IP) and immunofluorescence (IF)

We performed IHC, western blotting and IP according to previously described methods [8]. Especially, the key preconditions of IP experiment are: 1. the basal loading amounts of total protein, which is directly extracted from cells, are equal in each group; 2. the antibodies used for IP and immunoblotting (IB) are of equal amounts in each group. Primary antibodies used were: anti-(P)RR antibody (Sigma-Aldrich, Allentown, PA, USA, catalog #HPA003156), anti-active β-catenin antibody (Cell Signaling, Technology, Danvers, MA, USA, catalog #8814), anti-c-Myc antibody (Abcam, Eugene, OR, USA, catalog #ab39688), anti-Wnt3 antibody (Abcam, Eugene, OR, USA, catalog #ab32249), anti-NEDD4L antibody (Cell Signaling Technology, Danvers, MA, USA, catalog #4013), anti-flag antibody (HuaAn Biotechnology, Hangzhou, China, catalog #0912-1) and anti-GAPDH antibody (HuaAn Biotechnology, Hangzhou, China, catalog #ET1601-4). GAPDH served as internal control. IF was performed as previously described [17]. Primary antibodies used were: anti-Wnt3 antibody (Thermo Fisher Scientific, Waltham, MA, USA, catalog #PA5-18,516) and the same anti-NEDD4L antibody that was used in western blotting. All these antibodies were used according to the instructions provided by manufactures.

### Cell culture, gene silencing, editing, and sequencing

The colon cancer DLD-1 and HCT116 cells were purchased from the American Type Culture Collection (ATCC, Manassas, VA, USA) and cultured based on previously described protocols [8]. Silencing of (P)RR and NEDD4L in cells with shRNA was conducted as described previously [8]. Sequence of (P)RR shRNA was: 5’- GCTTCTAAGATCCTTGTTGACGCTCTGCATTCAAGAGATGCAGAGCGTCAACAAGGATCTTAGAAGCTTTTTT-3’. Sequence of NEDD4L shRNA was::5’-GCGAGTACCTATGAATGGATT-3’. *NEDD4L* knockout in DLD-1 cells was performed with TrueGuide™ NEDD4L sgRNA (Thermo Fisher Scientific, Waltham, MA, USA, catalog #A35533) using Crispr/Cas9 gene editing system according to the protocol described previously [18]. This sgRNA was specifically designed for *NEDD4L* gene knockout. Negative control was prepared using TrueGuide™ sgRNA Negative Control (Thermo Fisher Scientific, Waltham, MA, USA, catalog #A35526). DLD-1 cells were firstly transfected with TrueGuide™ NEDD4L sgRNA and TrueCut™ Cas9 Protein v2 (Thermo Fisher Scientific, Waltham, MA, USA, catalog #A36496) using the Lipofectamine™ CRISPRMAX™ Transfection Reagent (Thermo Fisher Scientific, Waltham, MA, USA, catalog #CMAX00001), according to the introductions of the reagents and protocol provided by the manufacture. Then single cells were sorted and seeded into single wells of a 96-well plate to form single cell clones. When we perform cell passage of these single cell clones (also called mono-colony), protein was simultaneously extracted from part of these cells for western blotting verification. According to the western blotting result, mono-colonies that showed relatively successful NEDD4L protein knockout were remained for the following passages. After several rounds of western blotting verification and mono-colony passages, the most stable deletion phenotype finally remained and thus the deletion types were purified during cell mitosis and passage processes. The finally remained cells from the purified mono-colony were used for the formal Sanger sequencing and western blotting experiments whose results are shown in the manuscript. Finally, Sanger sequencing-based editing efficiency analysis was made in cells from the purified mono-colony. Sanger sequencing is widely used in lots of published papers to sequence short pieces of DNA with specifically designed primers targeted on the designated gene. Primers for sequencing *NEDD4L* are: 5’-TAGCCTCAGCTCGCCAACAGTA-3’ (forward) and 5’-GGAGTTGTAAGGTGATGGCTGTG-3’ (reverse). The brief steps of Sanger sequencing are: 1. Amplify the specific gene by polymerase chain reaction (PCR) with chain-terminating AGCT dideoxyribonucleotides (ddNTPs) labeled by different fluorescence, so that DNA pieces with different sizes and fluorescence labels formed; 2. Separate DNA pieces with different sizes by using capillary gel electrophoresis; 3. Excite the fluorescence by laser and detect the different AGCT fluorescence signals by sequencing machine, thus the gene sequence is decoded [19]. After the gene-editing efficiency was determined in a pooled cell population, single cell clones with successful *NEDD4L* knockout were isolated for the following experiments.

### Cell wound healing, migration, and invasion

Migration ability of cells was evaluated via wound healing and trans-well migrating assays as previously described [20, 21]. Invasion ability of cells was detected through trans-well invading experiment as previously described [21].

### *Real-time polymerase chain reaction (PCR) and mRNA fluorescence *in situ* hybridization (FISH)*

We performed real-time PCR according to the previously described protocol [8]. Primer sequences for specific human genes are as follows: *ATP6AP2* ((P)RR encoding gene) (forward: ggcgttggtggcgggtgtyy; reverse: agcccatggacaatgcagccac) and 18S rRNA (forward: ggccctgtaattggaatgagtc; reverse: ccaagatccaactacgagctt). Human 18S rRNA was used as internal control. mRNA FISH with cells was performed as previously described [22], primer sequence for (P)RR was 5’- AGGTTATAGGGACTTGCTGGGTTCTTCGCTTG-3’.

### Heterozygous gastrointestinal epithelium-specific ATP6AP2 knock-in (ATP6AP2 WT/KI) mice

All the animal experiments were conducted in accordance to guidelines for the welfare and use of animals in cancer research established by The Second Affiliated Hospital, Zhejiang University School of Medicine. All procedures were approved by the Ethics Committee of The Second Affiliated Hospital, Zhejiang University School of Medicine (approval number: 202006). We generated a kind of gene-edited C57BL/6 mice with *ATP6AP2* knock-in in gastrointestinal epithelium by using Crispr/Cas9 technique. Generation of these mice was under the assistance of the GemPharmatech Company (Nanjing, China) and detailed procedures are shown in Additional file 1: Fig. S2. The control wildtype C57BL/6 mice were also purchased form the GemPharmatech Company (Nanjing, China). Mice were sacrificed at the age of 8 weeks, and their guts were resected, paraffin-embedded and sent for IHC as described previously [23].

### Gut microbiota analysis

Feces from *ATP6AP2* ^WT/KI^ and wildtype mice were collected and sent for gut microbiota 16S rRNA analysis according to the previously established protocols [24].

### Three-dimensional (3D) protein structure prediction

Prediction of 3D-protein structure of (P)RR, Wnt3, NEDD4L and Ub was performed online (https://www.swissmodel.expasy.org) according to the introduction on the website.

### Bioinformatic analysis

Bioinformatic analyses of the big data about gene transcripts, protein expression and gut flora distribution were conducted as previously described [25, 26].

### Statistical analysis

Statistical analyses were carried out using Prism software (GraphPad Software, La Jolla, CA, USA). Unpaired t test and one-way ANOVA followed by Tukey’s test were respectively conducted to compare numerical data between two groups and among multiple groups. Microbiota difference was analyzed using Wilcoxon rank-sum test. *P* < 0.05 was regarded as significantly different.

## Results

### Increased (P)RR level is associated with CRC progression

**Fig. 1:**
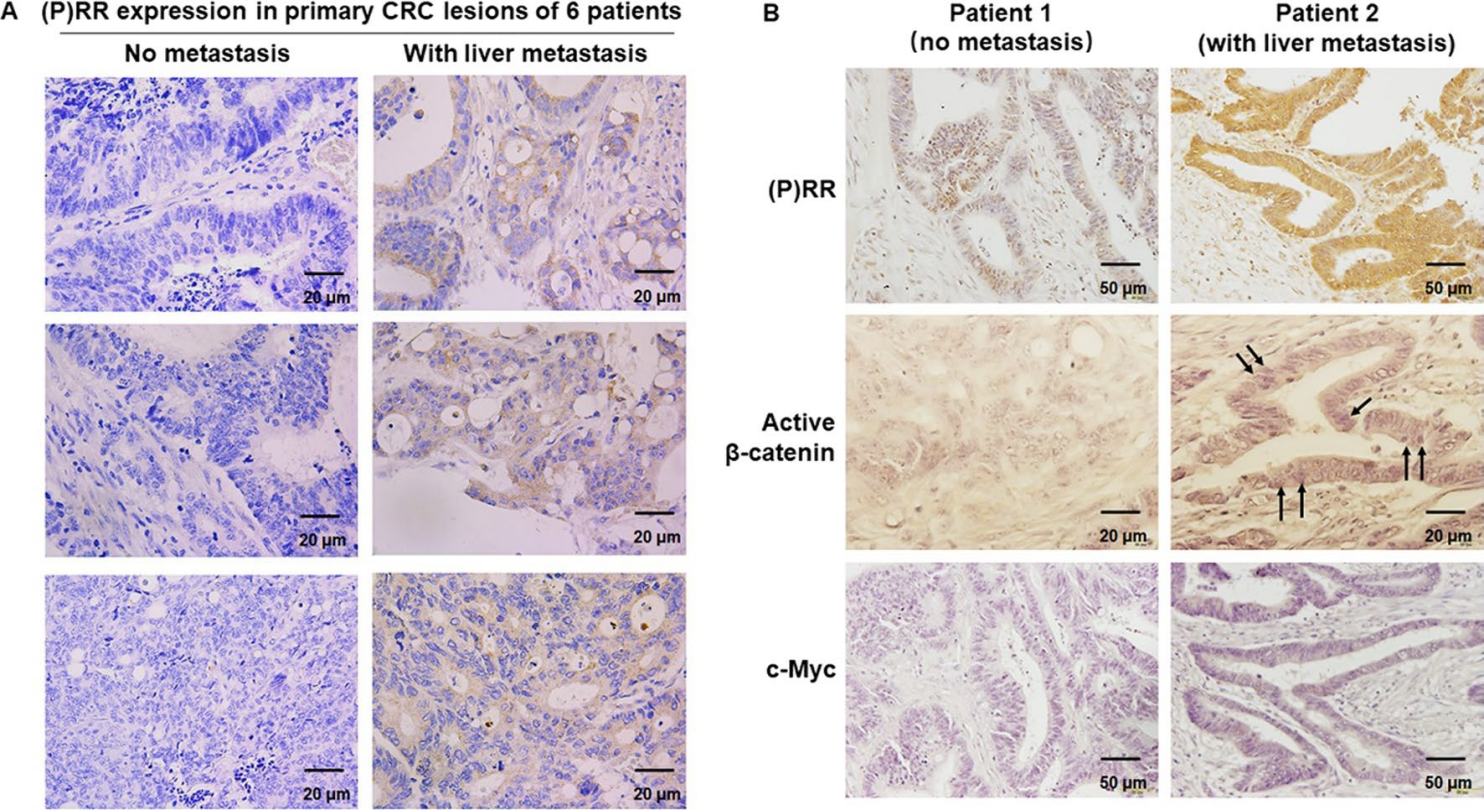
(P)RR and other Wnt signaling components are up-regulated in primary CRC lesions of patients with liver metastasis. **A** Representative IHC staining images of (P)RR in the primary CRC lesions of patients with liver metastasis or without metastasis. **B** Representative IHC staining images of (P)RR, as well as the downstream Wnt signaling components, active β-catenin and c-Myc, in the primary CRC lesions of individuals with liver metastasis or without metastasis. Brown color indicates positive protein staining. Black arrows indicate active β-catenin that translocated into cell nucleus

**Fig. 2:**
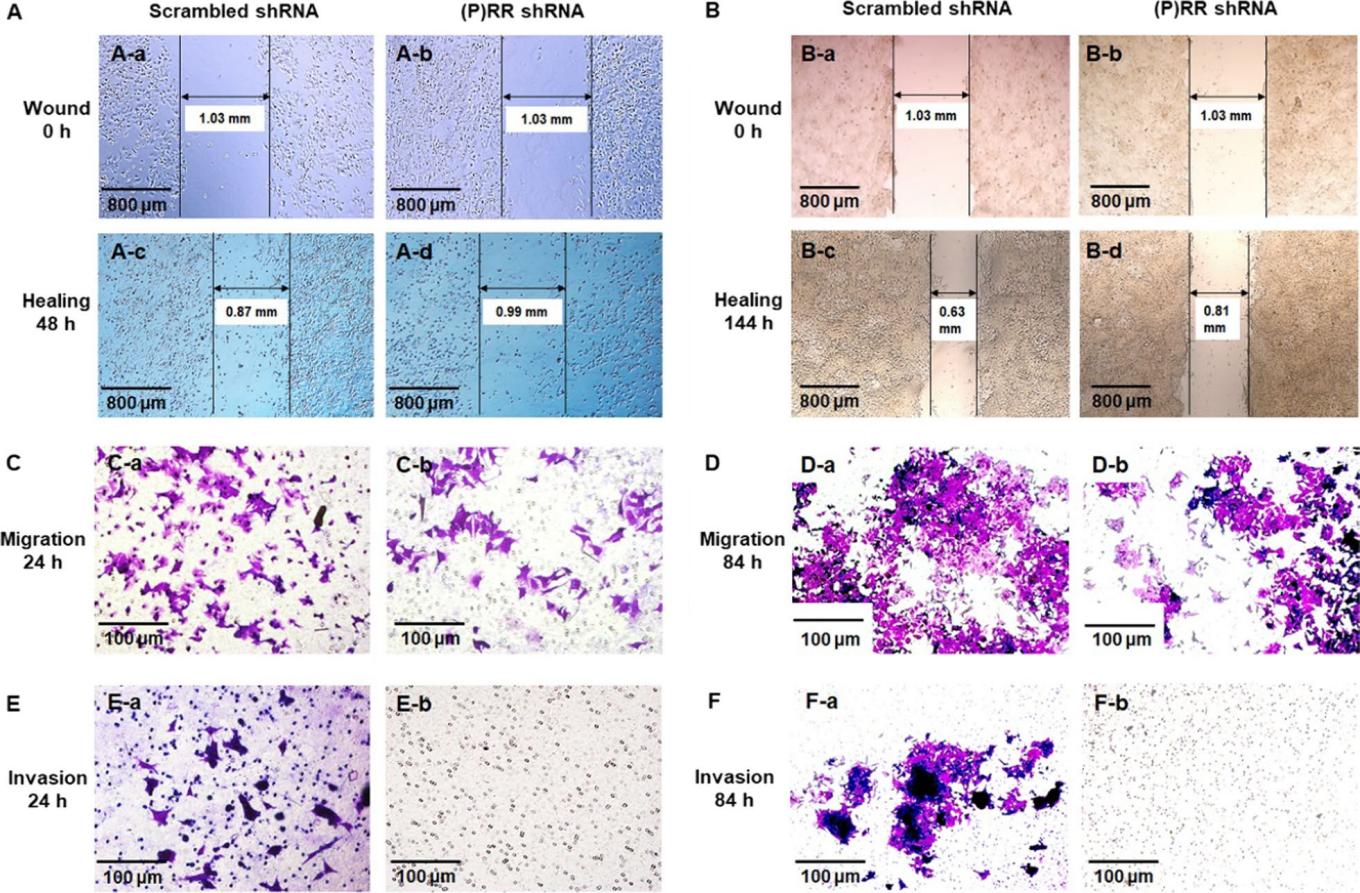
(P)RR silencing attenuates migrating and invading abilities of colon cancer cells. **A, B** Representative images showing the wound healing activity (reflects cell migrating ability) of DLD-1 (**A**) and HCT116 (**B**) with or without (P)RR silencing. A wound was made on the single layer of cells, followed by respective transfection with scrambled shRNA (**A-a, B-a**) and (P)RR shRNA (**A-b, B-b**) into cells. Wound healing degrees of DLD-1 (**A-c, d**) and HCT116 (**B-c, d**) were respectively observed at 48 h and 144 h after shRNA transfection. **(C, D)** Representative images showing the migration in trans-wells of DLD-1 (**C**) and HCT116 (**D**) with or without (P)RR silencing. **E**, **F** Representative images showing the invasion through the matrigel paved on the bottom of trans-wells of DLD-1 (**E**) and HCT116 (**F**) with or without (P)RR silencing

Firstly, we detected the protein levels of (P)RR as well as active β-catenin and c-Myc, the downstream components of Wnt signaling, in primary CRC lesions of patients with liver metastasis or without metastasis. Compared with that of patients without metastasis, (P)RR protein level is much higher in the primary CRC lesions of patients with liver metastasis (Fig. 1A). Furthermore, along with the increased (P)RR expression, more elevated protein levels of active β-catenin and c-Myc are also shown in primary CRC lesions of patients with liver metastasis than those of patients without metastasis (Fig. 1B). Considering the tumor tissues used consist of not only epithelial-derived cancer cells but also various interstitial cells, and it is impossible to extract protein purely from cancer cells out of interstitial cells for protein detection, western blotting was not performed with these samples. Next, we tried to make clear whether (P)RR directly affects migration and invasion of CRC cells, which are considered as basic cellular pathological behaviors of cancer progression. We induced (P)RR silencing in two colon cancer cell lines, DLD-1 and HCT116. Data show that (P)RR silencing dramatically inhibits the wound healing (reflects cell migration) (Fig. 2A, B), direct migrating (Fig. 2C, D) and invading (Fig. 2E, F) abilities of DLD-1 and HCT116. Therefore, elevated (P)RR might contribute to CRC progression by strengthening the progressive abilities of CRC cells, which could be partially owing to the increased Wnt signaling activity.

### (P)RR and Wnt3 are positively associated at protein level rather than mRNA level

**Fig. 3:**
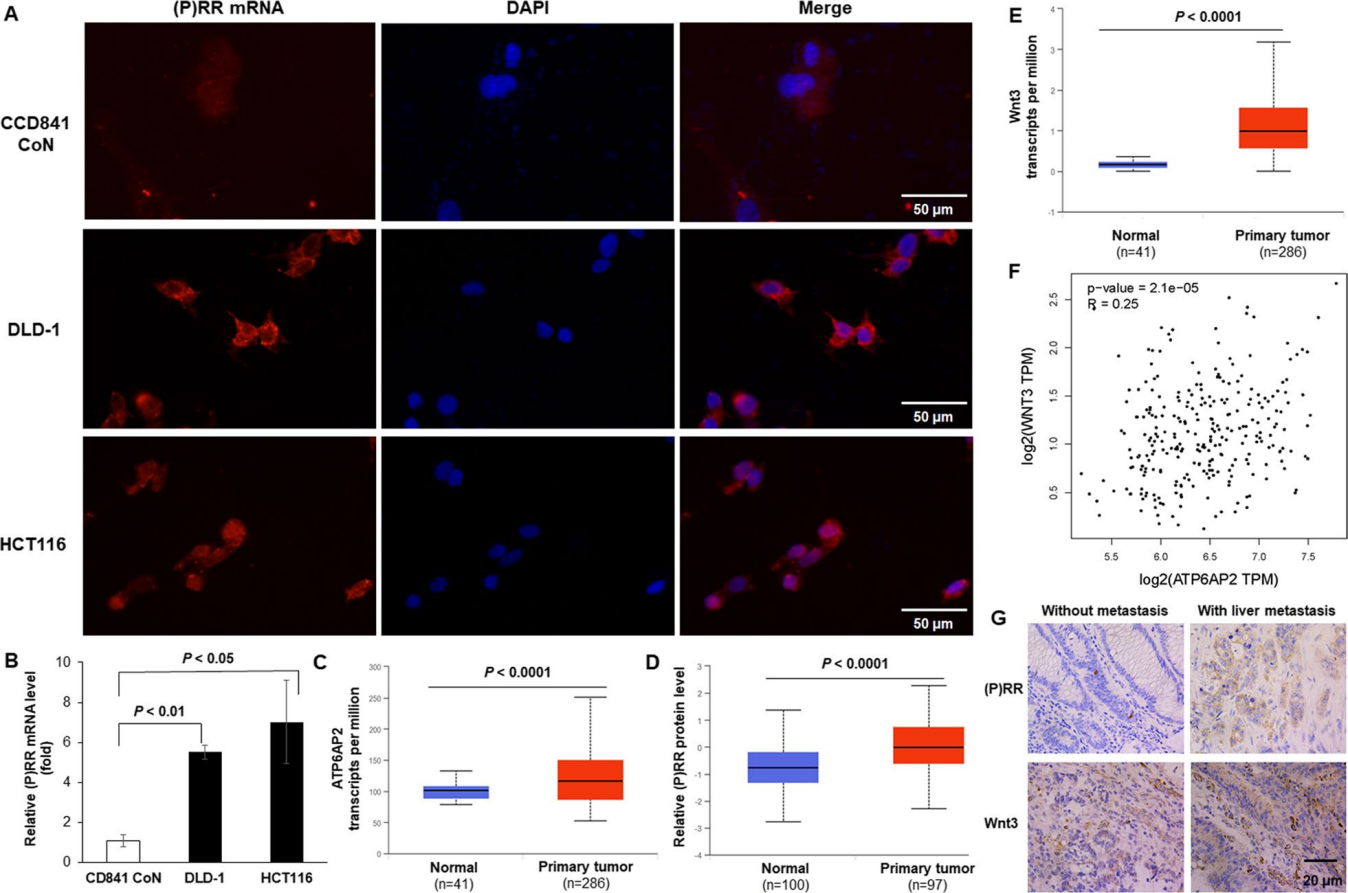
Up-regulated (P)RR and Wnt3 are positively associated at protein level in CRC. **A**, **B** (P)RR mRNA levels in CCD841 CoN, DLD-1 and HCT116 detected by mRNA FISH (**A**) and real-time PCR (n = 3 in each group) (**B**). **C** Transcriptional levels of *ATP6AP2* in primary colon cancer lesions and paracancerous normal tissues based on the TCGA database. **D** (P)RR protein levels in primary colon cancer lesions and paracancerous normal tissues based on the CPTAC database. **E** Transcriptional levels of *Wnt3* in primary colon cancer lesions and paracancerous normal tissues based on the TCGA database. **F** Little association is found between the transcriptional levels of *ATP6AP2* and *Wnt3* in colon cancer (r = 0.25). **G** Representative IHC staining images of (P)RR and Wnt3 in the primary CRC lesions of individuals with liver metastasis or without metastasis. Brown color indicates positive protein staining

WE previously revealed that (P)RR protein level is higher in colon cancer cells than that in normal colon epithelial cells [8], however, the situation at transcriptional level remains unclear. In the present study, we performed mRNA FISH (Fig. 3A) and real-time PCR (Fig. 3B) to see the respective amount of (P)RR mRNA in normal colon epithelial cell line CCD841 CoN, as well as DLD-1 and HCT116. Data show that (P)RR mRNA level is significantly higher in DLD-1 and HCT116 compared with that in CCD841 CoN, in both relative amount (Fig. 3A) and absolute quantification (Fig. 3B). By analyzing the bioinformatic data of human tissue samples provided in The Cancer Genome Atlas (TCGA) (Fig. 3C) and Clinical Proteomic Tumor Analysis Consortium (CPTAC) (Fig. 3D) databases, we found consistantly elevated levels of *ATP6AP2* ((P)RR encoding gene) transcripts (Fig. 3C) and (P)RR protein (Fig. 3D) in primary lesions of colon cancer in comparison to those of paracancerous normal tissues. Based on the TCGA dataset analysis, higher transcriptional level of *Wnt3* is found in primary lesions of colon cancer in comparison to that of paracancerous normal tissues (Fig. 3E). However, there is little association between the transcriptional levels of *ATP6AP2* and *Wnt3* in colon cancer (r = 0.25) (Fig. 3F). Additionally, primary CRC lesions of patients with liver metastasis show higher protein levels of both (P)RR and Wnt3, compared with those of CRC patients without metastasis or only with peritoneum metastasis (Fig. 3G, Additional file 1: Fig. S1A). These data are in agreement with our previous finding that (P)RR-downregulation attenuates Wnt3 protein level without affecting *Wnt3* mRNA level in DLD-1 and HCT116 [8]. To summarize, (P)RR and Wnt3 are up-regulated in CRC especially that with progression, moreover, (P)RR and Wnt3 are positively associated at protein level rather than mRNA level.

### NEDD4L mediates the (P)RR downregulation-induced Wnt3 ubiquitination in CRC.

WE previously demonstrated that (P)RR silencing decreases Wnt3 protein without affecting *Wnt3* mRNA level in DLD-1 and HCT116 [8]. However, detailed mechanisms by which (P)RR regulates Wnt3 protein remain unknown. Considering the fact that (P)RR silencing affects Wnt3 protein at the post-translational level, we speculate the influenced step may lie in protein ubiquitination and subsequent degradation. As explained in the introduction part, NEDD4L should be a possible E3 ubiquitin ligase that mediates the (P)RR silencing-induced Wnt3 ubiquitination. Hereinafter, we made the efforts to verify these hypotheses.

**Fig. 4:**
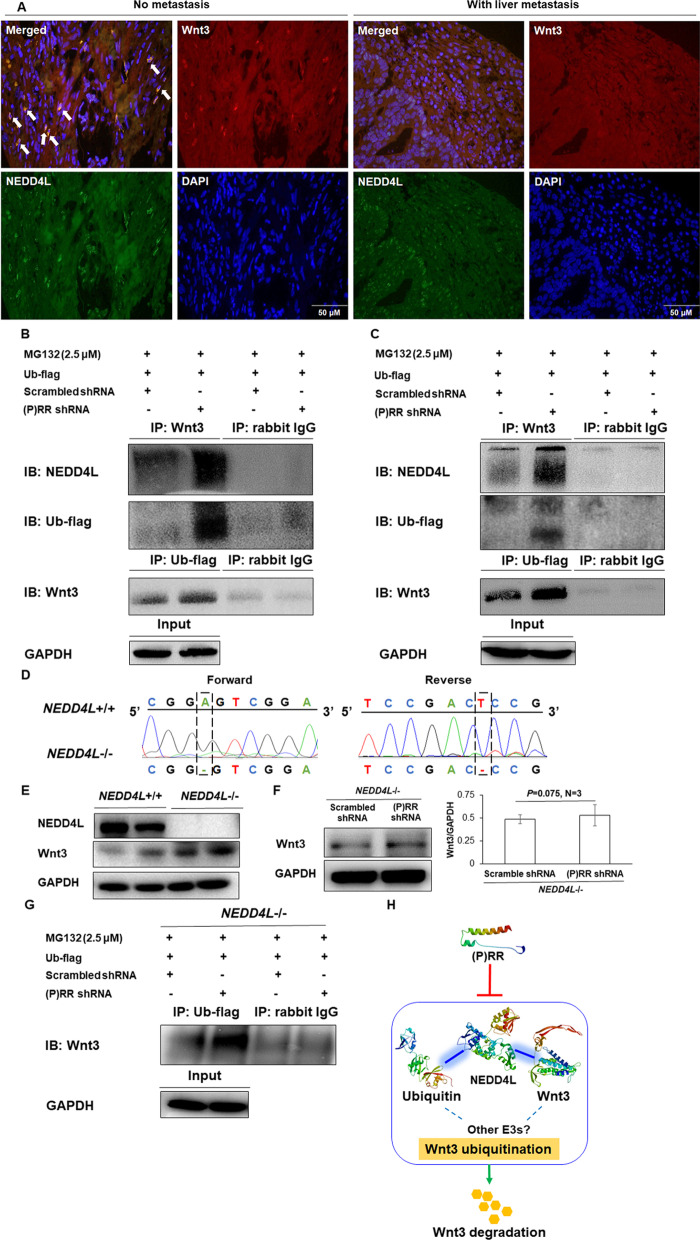
NEDD4L mediates the (P)RR downregulation-induced Wnt3 ubiquitination. **A** Representative images of IF detecting the Wnt3-NEDD4L colocalization in primary CRC lesions of individuals with liver metastasis or without metastasis. Red color indicates Wnt3 protein staining. Green color indicates NEDD4L protein staining. Blue color indicates nulus stained by DAPI. Yellow color indicated by white arrows represents the merged red and green colors, namely Wnt3-NEDD4L colocalization. DAPI: 4',6-diamidino-2-phenylindole. **B**, **C** Representative images of IP detecting the binding levels of Wnt3-Ub and Wnt3-NEDD4L in DLD-1 (**B**) and HCT116 (**C**) with or without (P)RR silencing. Ub marked by a flag label was transfected into cells to make the small molecule Ub more easily to be detected. Ahead of IP, a proteasome inhibitor MG132 was used to inhibit the possible degradation of Wnt3 triggered by ubiquitination. **D** DNA sequencing result of DLD-1 with NEDD4L knockout (DLD ^NEDD4L−/−^). **E** Representative images of western blotting detecting the protein levels of NEDD4L and Wnt3 in wildtype DLD-1 (DLD ^NEDD4L+/+^) and DLD ^NEDD4L−/−^. **F** Representative images of western blotting detecting the Wnt3 protein levels in DLD ^NEDD4L−/−^ with or without (P)RR silencing and the quantification of band intensity. **G** Representative images of IP detecting the Wnt3-Ub binding level in DLD ^NEDD4L−/−^ with or without (P)RR silencing. Ub marked by a flag label was transfected to cells to make the small molecule Ub more easily to be detected. Ahead of IP, a proteasome inhibitor MG132 was used to inhibit the possible degradation of Wnt3 triggered by ubiquitination. **H** Molecular mechanism by which (P)RR affects Wnt3 protein level. (P)RR inhibits the formation of Wnt3-NEDD4L-Ub complex, thus attenuates the NEDD4L-mediated Wnt3 ubiquitination and subsequent Wnt3 degradation. However, NEDD4L may not be the only E3 that mediates the (P)RR silencing-induced Wnt3 ubiquitination

Firstly, we detected the colocalization, which indirectly reflects possible protein–protein binding, of Wnt3 and NEDD4L proteins in primary CRC lesions of patients without metastasis or with liver metastasis. As representative photos shown in Fig. 4A, there is hardly any Wnt3-NEDD4L colocalization displayed in primary CRC lesions of patients with liver metastasis, which means Wnt3 protein is more likely to be protected from the possible NEDD4L-mediated ubiquitination. In contrast, much more obovious Wnt3-NEDD4L colocalization is seen in primary CRC lesions of patients without metastasis, which increased the possibility of NEDD4L-mediated Wnt3 ubiquitination.

To investigate the potential direct interaction among Wnt3, Ub and NEDD4L, we performed IP. Ahead of IP, DLD-1 and HCT116 cells were treated with a proteasome inhibitor MG132 to inhibit the possible Wnt3 degradation triggered by ubiquitination. We treated cells with equal amounts of MG132 in each group. If MG132 could completely inhibit the (P)RR silencing-induced Wnt3 protein degradation, then bait Wnt3 protein levels in each group should be equal since the loading amounts of total protein were equal. If MG132 could not completely inhibit the (P)RR silencing-induced Wnt3 protein degradation, then bait Wnt3 protein level might be slightly lower in (P)RR silencing group than (P)RR non-silencing group. Therefore, the bait Wnt3 protein level in (P)RR silencing group should be no more than (equal to or less than) that in (P)RR non-silencing group. We found that (P)RR silencing induces dramatically increased binding levels of both Wnt3-NEDD4L and Wnt3-ubiquitin (Fig. 4B, C) suggesting that NEDD4L mediates the (P)RR silencing-induced Wnt3 ubiquitination. This is consistent with the existing knowledge about NEDD4L as a CRC suppresor. As supportive information, the analyzing results of public big data indicate decreased NEDD4L in primary CRC lesions at both transcriptional (Additional file 1: Fig. S1B) and protein (Additional file 1: Fig. S1C) levels in comparison to the paracancerous normal tissues. Therefore, overexpressed (P)RR may inhibit the NEDD4L-mediated Wnt3 ubiquitination, thus lead to declined Wnt3 degradation as well as consequent increased Wnt3 protein and Wnt signaling activity in CRC.

Next, we asked two additional questions: Whether NEDD4L knockout could ameliorate the Wnt3 down-regulation induced by (P)RR silencing? Whether NEDD4L is the only E3 ubiquitin ligase that mediates Wnt3 ubiquitination induced by (P)RR silencing? To answer these questions, we constructed a DLD-1 cell line with *NEDD4L* gene knockout (DLD-1^*NEDD4L*−/−^) using Crispr/Cas9 system. Since the human *NEDD4L* gene is only located on chromosome 18 rather than other chromosomes [27], Sanger sequencing with the specifically designed primers targeted on *NEDD4L* gene was perfomed to varify the successful *NEDD4L* knockout. DNA sequencing confirmed that a base A/T was successfully deleted from the *NEDD4L* gene (Fig. 4D), which led to failed expression of NEDD4L protein in DLD-1^*NEDD4L*−/−^ cells (Fig. 4E). Moreover, Wnt3 protein level remarkably increases in DLD-1^*NEDD4L*−/−^ cells (Fig. 4E). In our previously published paper, we have already demonstrated that (P)RR silencing in wildtype DLD-1 causes decreased Wnt3 protein level without affecting the *Wnt3* mRNA level [8]. However, when (P)RR silencing is induced in DLD-1^*NEDD4L*−/−^ cells, Wnt3 protein level is not accordingly decreased as the situation of wildtype DLD-1 cells (Fig. 4F). Therefore, NEDD4L knockout ameliorates the effect of (P)RR silencing on Wnt3 down-regulation. In other words, the degradation-promoting effect of (P)RR silencing on Wnt3 protein closely depends on the existence of NEDD4L. We also performed IP with DLD-1^*NEDD4L*−/−^ cells after MG132 treatment. As shown in Fig. 4G, we found that (P)RR silencing still slightly increases Wnt3-ubiquitin binding level in DLD-1^*NEDD4L*−/−^ cells, suggesting there may be other E3(s) that also mediate(s) Wnt3 ubiquitination and influenced by (P)RR. Collectively, (P)RR inhibits the formation of Wnt3-NEDD4L-Ub complex, thus attenuates the NEDD4L-mediated Wnt3 ubiquitination and subsequent Wnt3 degradation (Fig. 4H). Moreover, NEDD4L could not affect Wnt3 protein level by itself without combining with (P)RR-downregulation. However, since *NEDD4L* knockout could not completely reverse the amplified Wnt3 ubiquitination caused by (P)RR silencing (Fig. 4G), NEDD4L should not be the only E3 that mediates this process (Fig. 4H).

In sumarry, overexpressed (P)RR in CRC inhibits the NEDD4L-mediated Wnt3 ubiquitination, thus protects Wnt3 protein from degradation, up-regulatesWnt3 protein and Wnt signaling activity, and finally promotes CRC progession.

### Mouse gastrointestinal epithelium with ATP6AP2 knock-in shows increased Wnt3 protein and diminished Wnt3-NEDD4L colocalization

**Fig. 5:**
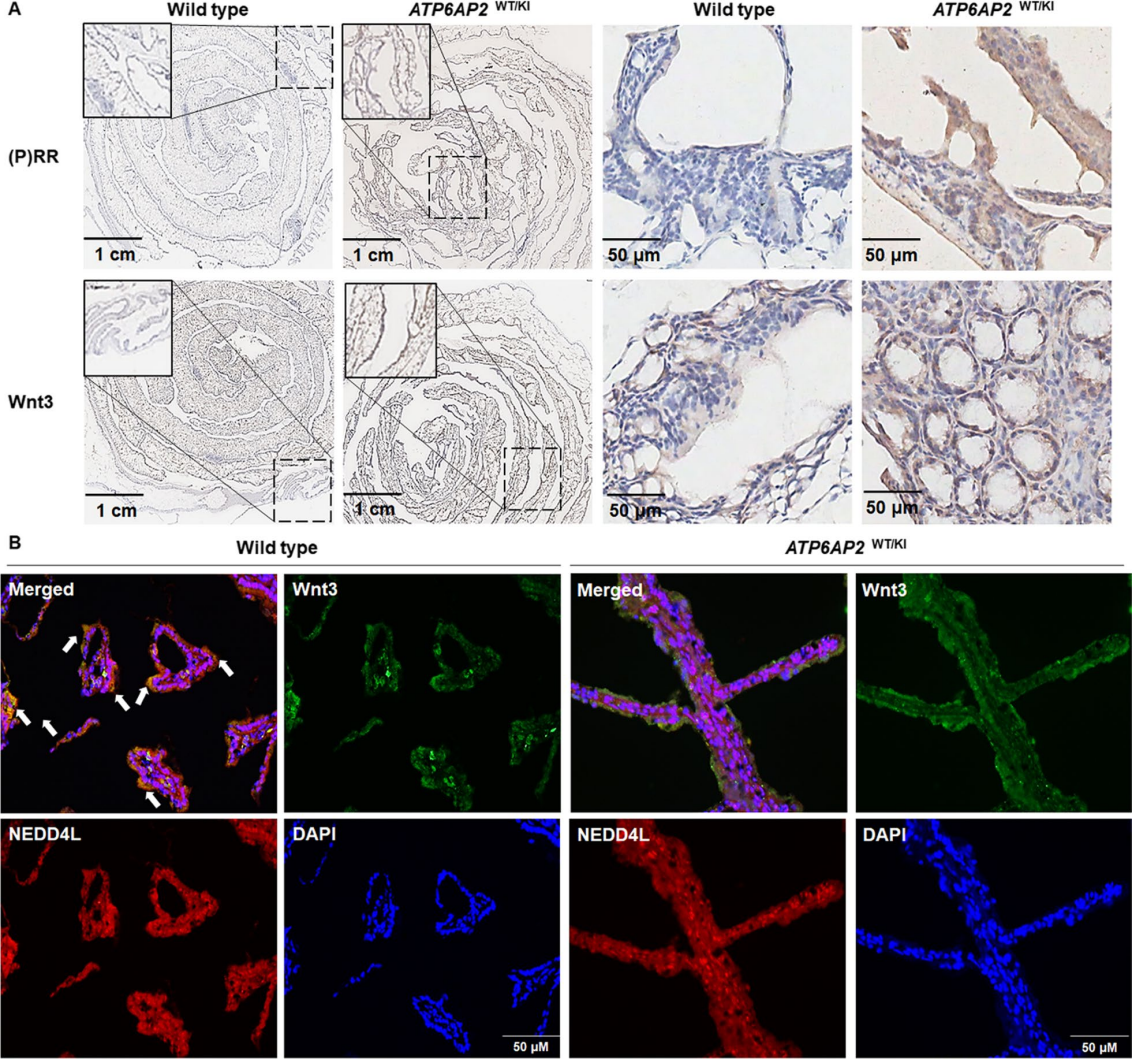
Wnt3 is up-regulated and Wnt3-NEDD4L colocalization is down-regulated in the gut epithelium of *ATP6AP2* ^WT/KI^ mice. **A** Representative IHC staining images of (P)RR and Wnt3 in the gut epithelium of *ATP6AP2* ^WT/KI^ mice and wildtype mice. **B** Representative images of IF detecting the Wnt3-NEDD4L colocalization in the gut epithelium of *ATP6AP2* ^WT/KI^ mice and wildtype mice. Red color indicates NEDD4L protein staining. Green color indicates Wnt3 protein staining. Blue color indicates nulus stained by DAPI. Yellow color indicated by white arrows represents the merged red and green colors, namely Wnt3-NEDD4L colocalization. DAPI: 4',6-diamidino-2-phenylindole

To deeply investigate the detailed physio-pathological function of (P)RR in vivo, we generated a kind of heterozygous gastrointestinal epithelium-specific *ATP6AP2* knock-in (*ATP6AP2* ^WT/KI^) mice by using Crispr/Cas9 system. Detailed strategy for generating this mouse model is shown in Additional file 1: Fig. S2. Compared with homologous wildtype mice, *ATP6AP2* knock-in not only induces (P)RR protein overexpression but also dramatically increases Wnt3 protein level in mouse gut epithelium (Fig. 5A), while Wnt3-NEDD4L colocalization is remarkably diminished in *ATP6AP2* ^WT/KI^ mice (Fig. 5B). Considering the tissues used consist of not only epithelial cells but also various interstitial cells, and it is impossible to extract protein purely from epithelial cells out of interstitial cells for protein detection, western blotting was not performed with these samples. In agreement with the in vitro findings described above, these in vivo data further support the speculation that (P)RR inhibits the NEDD4L-mediated Wnt3 ubiquitination and subsequent degradation, so that increases Wnt3 protein level.

### (P)RR overexpression in gastrointestinal epithelium modulates gut microbiota.

**Fig. 6:**
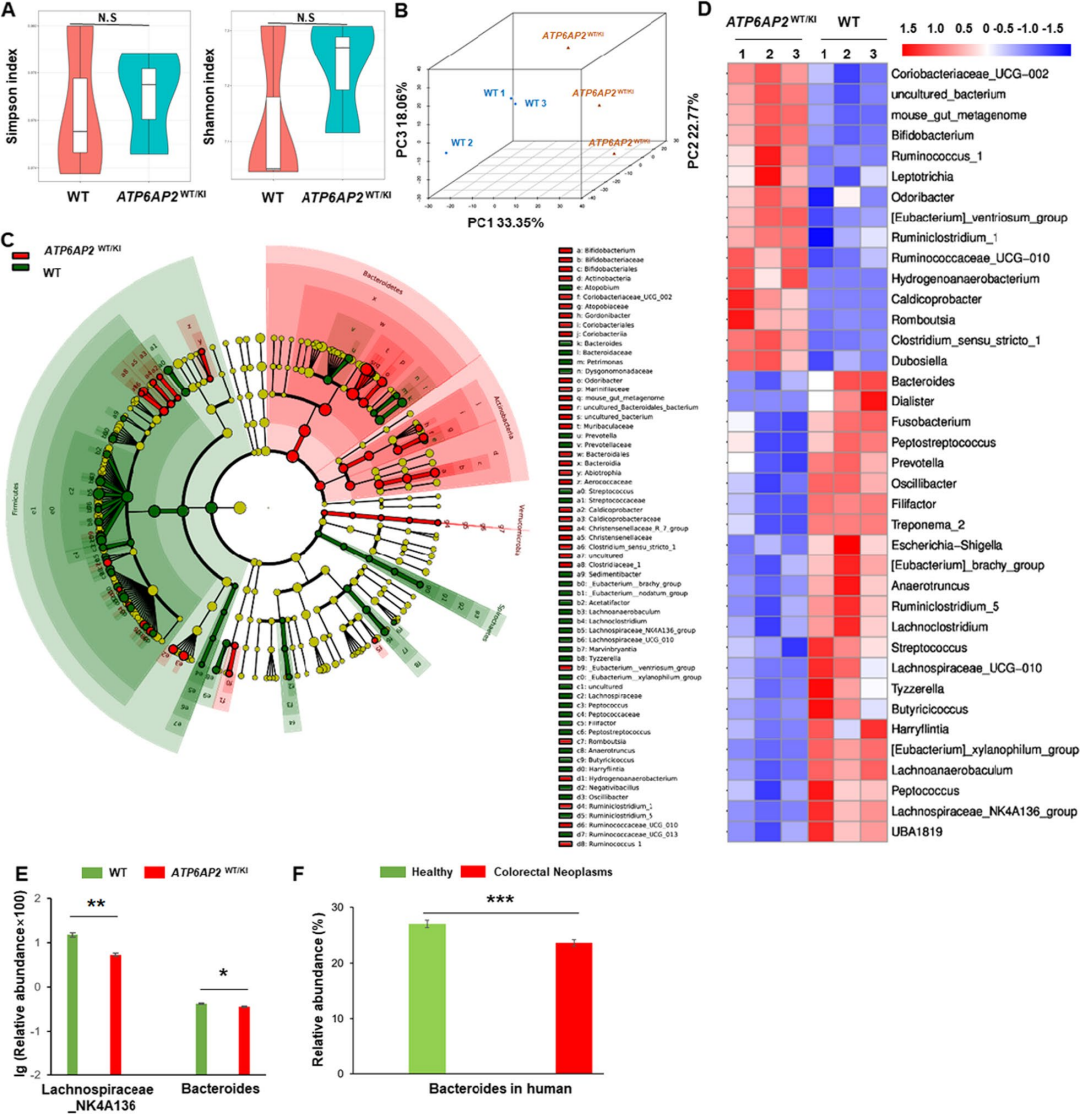
(P)RR overexpression in gastrointestinal epithelium modulates gut microbiota. **A**, **B** Alpha (**A**) and beta (**B**) diversity of gut microbiota in *ATP6AP2* ^WT/KI^ mice and wildtype (WT) mice. **C** Cladogram of gut microbiota in *ATP6AP2* ^WT/KI^ mice and WT mice. Red nodes indicate flora with significantly higher abundance in *ATP6AP2* ^WT/KI^ mice than WT mice. Green nodes indicate flora with significantly higher abundance in WT mice than *ATP6AP2* ^WT/KI^ mice. Yellow nodes represent flora that did not differ significantly between the two groups. The diameter of a node is proportional to the relative flora abundance. Each layer of nodes represents a gate/class/order/family/genus from the inside out. Notes for each layer of flora indicate phylum/class/order/family/genus from the outside in. **D** Heatmap of genus with significantly different abundance in the two groups. Red indicates relatively high genus abundance and blue indicates relatively low genus abundance. Scales on the scalebar mark relative abundances of microbiota. **E** Relative abundance of Lachnospiraceae_NK4A136 and Bacteroides genus in *ATP6AP2* ^WT/KI^ mice and wildtype mice (n = 3 in each group; *P < 0.05, **P < 0.01). **F** Relative abundance of Bacteroides genus in CRC patients and healthy controls based on the GMrepo database (n = 1000 in each group; ***P < 0.001)

Given that CRC progression is regulated by not only the gene state of cancer cells but also gut microbiota. We also investigated whether (P)RR overexpression in gastrointestinal epithelium of *ATP6AP2* ^WT/KI^ mice affects gut flora distribution and the abundance of specific CRC-related bacteria genus. We analyzed and compared the gut microbiota states of wildtype and *ATP6AP2* ^WT/KI^ mice, and found similar alpha-diversity within each group (Fig. 6A) but different beta-diversity between the two groups (Fig. 6B). We also detected bacteria gates/classes/orders/families/genus that are different or similar between the two groups (Fig. 6C) and listed the representative genus with significantly different abundance between the two groups (Fig. 6D). Notably, *ATP6AP2* knock-in significantly decreases the relative abundance of Lachnospiraceae_NK4A136 genus (Fig. 6E), which is known to be positively associated with anti-tumor immune factors, intestinal barrier-related factors, and anti-inflammatory response [28]. Additionally, *ATP6AP2* knock-in also leads to the shrink of Bacteroides genus (Fig. 6E), the relative abundance of which is prevalent (20–30% among all genus) in human. Consistently, Bacteroides genus is found decreased in patients with colorectal neoplasm than healthy controls by analyzing the public big data from the Data Repository for Human Gut Microbiota (GMrepo) database (Fig. 6F).

## Discussion

The present study reveals, for the first time, that (P)RR promotes CRC progression through inhibiting the NEDD4L-mediated Wnt3 ubiquitination and modulating gut microbiota. Three novel findings are established: First, higher (P)RR expression in progressive CRC enhances migrating and invading abilities of CRC cells, which should be partially owing to the increased Wnt signaling activity; Second, (P)RR in CRC inhibits the NEDD4L-mediated Wnt3 ubiquitination, thus protects Wnt3 protein from degradation; Third, (P)RR modulates gut microbiota, especially, (P)RR decreases the abundances of Lachnospiraceae_NK4A136 and Bacteroides genus which are considered as protective bacteria against CRC progression.

We have previously suggested that aberrant (P)RR expression contributes to CRC oncogenesis and associates with poor prognosis of patients [8]. In this study, we further indicate that (P)RR protein level is much higher in the primary lesions of progressive CRC than non-progressive CRC, which also leads to increased Wnt3 protein and Wnt signaling activity in progressive CRC. Additionally, this study provides direct evidence that (P)RR plays an essential role in maintenance of the crucial cell abilities related to cancer progression as migration and invasion. Therefore, (P)RR promotes not only CRC oncogenesis but also CRC progression.

In the past decades, most studies about Wnt ligands were mainly focused on their synthesis, processing, secretion, and reception [29, 30], while little knowledge of Wnt protein degradation has been reported in detail. In the present study, we indicate that the E3 ubiquitin ligase NEDD4L plays a pivotal role in mediating Wnt3 ubiquitination in CRC. Furthermore, (P)RR plays an inhibitory role on the NEDD4L-mediated Wnt3 ubiquitination, thus protects Wnt3 from degradation triggered by ubiquitination. Quadri et al. [13] previously suggested that (P)RR attenuates the protein ubiquitination-catalyzing activity of NEDD4L by inducing NEDD4L phosphorylation, which should be supportive and complementary to our findings. It has been verified that the upstream signal of Wnt/β-catenin pathway is critically essential for Wnt signaling activity, despite of the existence of constitutive pathway component mutations [8, 31]. Therefore, Wnt3 protein that protected by (P)RR and escaped from degradation will further augment Wnt signaling activity, and finally promotes CRC progression.

Mounting recent evidence have revealed the prevalence of pathological microbial imbalance or dysbiosis in the guts of CRC patients. In the present study, we analyzed the effect of gastrointestinal-specific *ATP6AP2* knock-in on mouse gut microbiota. Our data show that *ATP6AP2* knock-in leads to (P)RR overexpression in gut epithelium, and this significantly affects bacterial flora distribution in mice gut. Particularly, (P)RR overexpression decreases the abundances of Lachnospiraceae_NK4A136 and Bacteroides genus that are generally thought to be protective against CRC. Lachnospiraceae_NK4A136 genus is considered as intestinal probiotics due to its positive association with intestinal barrier-related factors, anti-tumor immune factors (IL-2, INF-g, and NK cells) and anti-inflammatory response [28, 32]. Bacteroides genus is a normal inhabitant of human intestine and accounts for about 30% of intestinal microbiota. Bacteroides genus maintains complex and generally beneficial influence on mucosal immune system development and intestinal homeostasis [33, 34]. Consistently, our analysis of big data in public databases also shows that the relative abundance of Bacteroides genus is generally reduced in feces of CRC patients in comparison to that of healthy controls. However, individual bacteria species of Bacteroides genus play controversial roles in CRC. For example, Bacteroides fragilis (B. fragilis) is an anaerobic Gram-negative bacterium colonizing about 0.5% to 2% of the whole human intestine [34]. Lee et al. [35] previously provided direct evidence that B. fragilis inhibits colitis-associated CRC by producing polysaccharide A (PSA) and further activating the Toll-like receptor 2 (TLR2) signaling in murine models. Interestingly, some studies suggested that B. fragilis includes two strains: the non-toxigenic B. fragilis that exerts protective effect against CRC oncogenesis and progression, and the toxigenic B. fragilis that favors cellular injury and CRC development. Therefore, further works should be required in the future to identify the specific bacteria species that are affected by (P)RR and associated with CRC progression. To summarize, the promotional contribution of (P)RR to CRC progression should be partially due to its inhibitory effect on Lachnospiraceae_NK4A136 and Bacteroides genus.

## Conclusions

Collectively, (P)RR promotes CRC progression through inhibiting the NEDD4L-mediated Wnt3 ubiquitination and modulating gut microbiota especially the down-regulation of Lachnospiraceae_NK4A136 and Bacteroides genus (Additional file 1: Fig. S3). These novel findings are prospective to be translated into effective therapeutic strategy of CRC in the future.

## Supplementary Information

**Additional file 1.** Expression of (P)RR, Wnt3 and NEDD4L in CRC.

## Data Availability

Materials supporting the conclusions of this paper have been included within this article.

## Abbreviations

(P)RR: (Pro)renin receptor
CRC: Colorectal cancer
FISH: Fluorescence in situ hybridization
ATP6AP2 WT/KI mice: Gastrointestinal epithelium-specific ATP6AP2 knock-in mice
EMT: Epithelial-mesenchymal transformation
IHC: Immunohistochemistry
IP: Immunoprecipitation
IF: Immunofluorescence
Ub: Ubiquitin
E3s: E3 ubiquitin ligases
NEDD4L: Neural precursor cell expressed developmentally downregulated gene 4-like
PSA: Polysaccharide A
TLR2: Toll-like receptor 2
TCGA: The cancer genome atlas
CPTAC: Clinical proteomic tumor analysis consortium
KD: Knockdown
Ub: Ubiquitin
IP: Immunoprecipitation
IB: Immunoblotting
DAPI: 4',6-Diamidino-2-phenylindole
ddNTP: Dideoxyribonucleotides
