# Supplementary material for: (Pro)renin receptor promotes colorectal cancer progression through inhibiting the NEDD4L-mediated Wnt3 ubiquitination and modulating gut microbiota
Source: Cell Commun Signal. 2023 Jan 3;21:2. doi: 10.1186/s12964-022-01015-x (PMC9809055; doi:10.1186/s12964-022-01015-x)
Supplement: Supplementary file 2 — Additional file 1. Expression of (P)RR, Wnt3 and NEDD4L in CRC. [file 12964_2022_1015_MOESM2_ESM.docx]

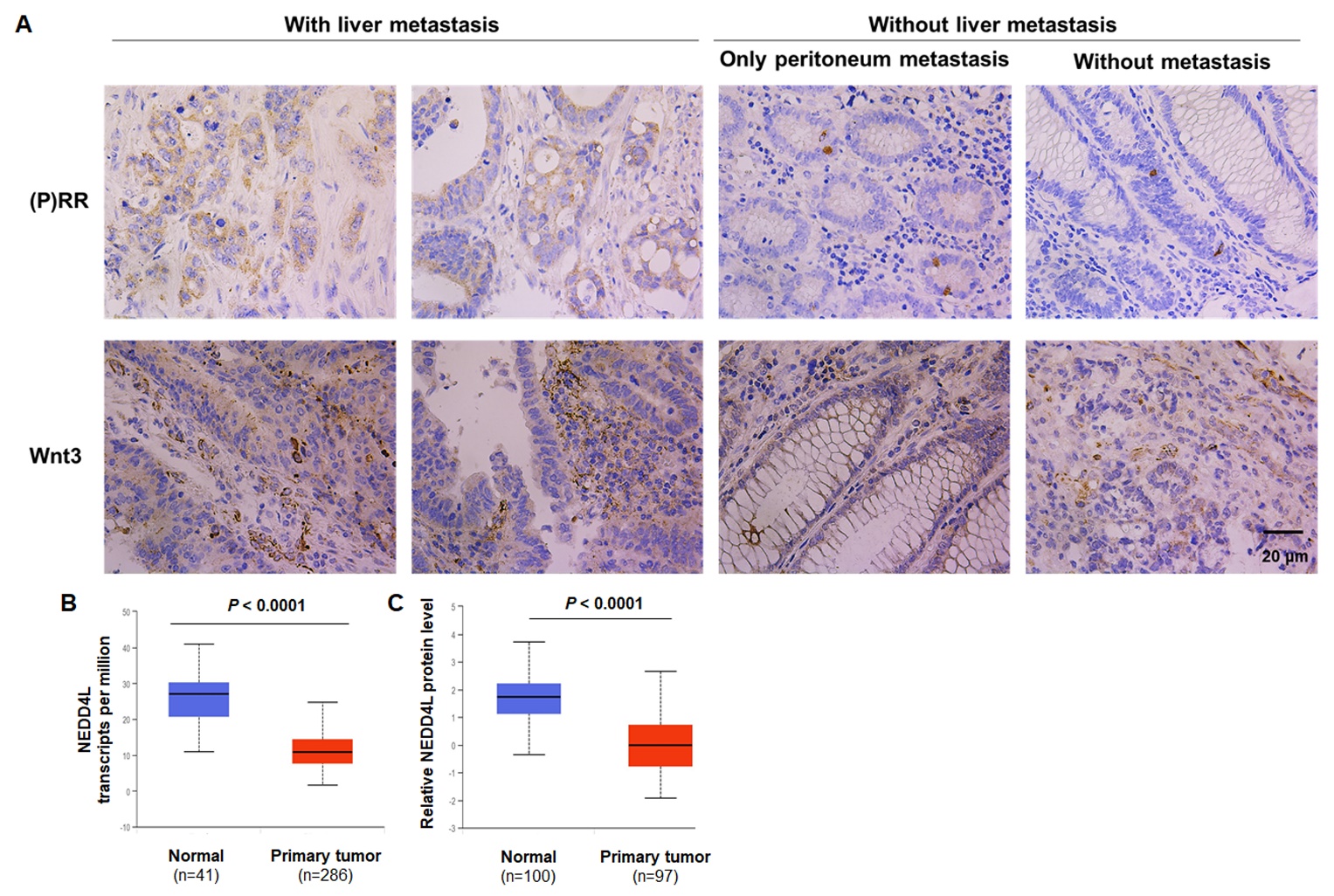


**Supplementary figure 1.** Expression of (P)RR, Wnt3 and NEDD4L in CRC. **(A)** Protein levels of (P)RR and Wnt3 are positively associated in primary CRC lesions. Brown color indicates positive protein staining. **(B)** Transcriptional levels of *NEDD4L* in primary colon cancer lesions and paracancerous normal tissues based on the TCGA database. **(C)** NEDD4L protein levels in primary colon cancer lesions and paracancerous normal tissues based on the CPTAC database.


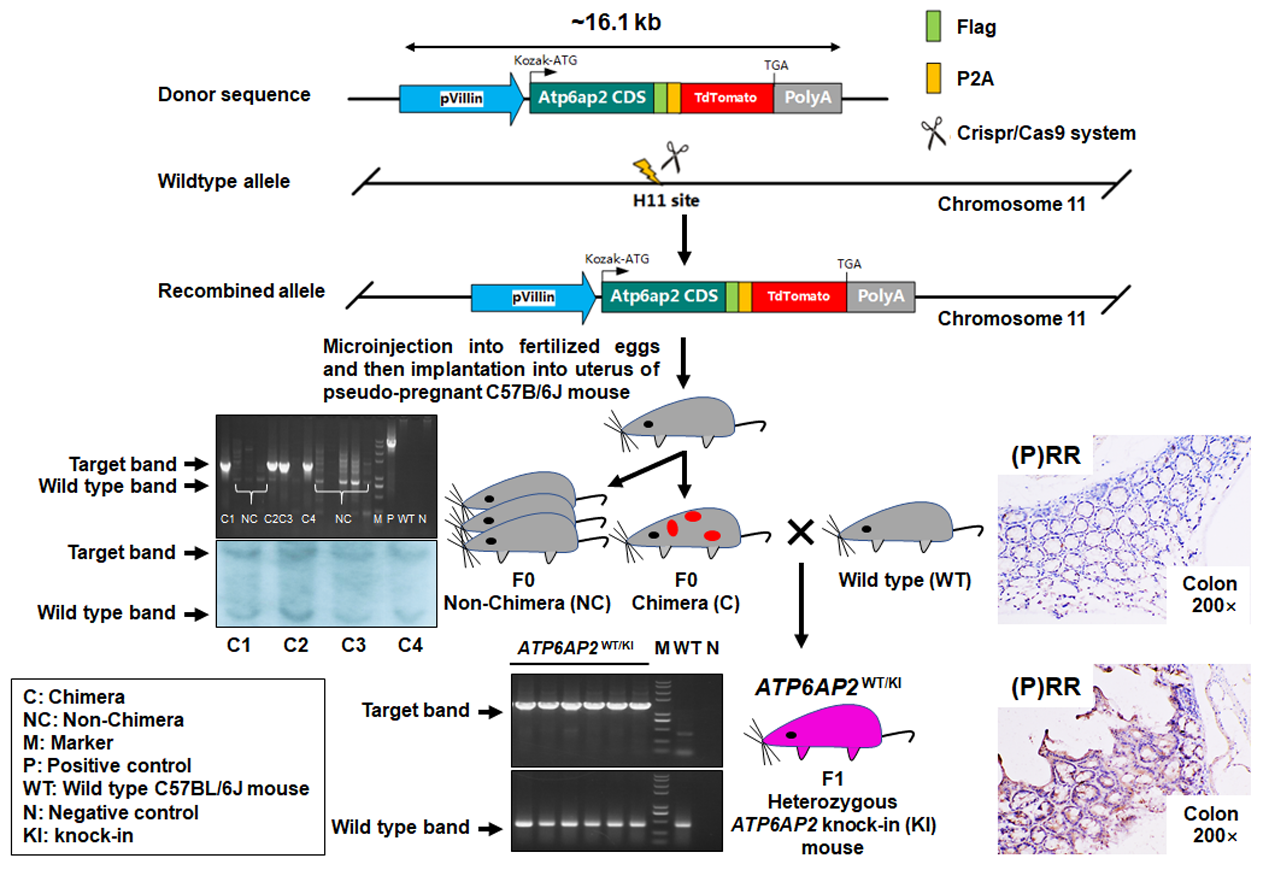


**Supplementary figure 2.** Process of generating the heterozygous gastrointestinal epithelium-specific *ATP6AP2* knock-in (*ATP6AP2* ^WT/KI^) mice. Firstly, A donor sequence containing an *ATP6AP2* coding sequence (CDS) in the middle, a Villin-dependent promoter sequence (*pVillin*) at the upstream and a TdTomato sequence at the downstream was built. Secondly, the donor sequence was inserted into the H11 site of a wildtype allele on Chromosome 11 using Crispr/Cas9 gene editing system to build a recombined allele. Next, the recombined allele was microinjected into fertilized eggs, followed by the implantation of the eggs into uterus of a pseudo-pregnant C57B/6J mouse to breed the mice of F0 generation. Genotyping was performed with the cut tails of F0 mice using PCR, southern blotting, and gene sequencing. Among the mice of F0 generation, there were both non-chimera (wildtype) mice and chimera mice that carrying the recombined allele (target band shown on the gel). Then we crossed chimera mice with wildtype mice to breed the heterozygous mice of F1 generation (*ATP6AP2* ^WT/KI^) that stably carrying a wild allele and a recombined allele with *ATP6AP2* knock-in. The heterozygous genotype of F1 generation was confirmed using PCR and gene sequencing. (P)RR protein overexpression in the gut epithelium of *ATP6AP2* ^WT/KI^ mice was confirmed by IHC. These *ATP6AP2* ^WT/KI^ mice were used for investigating the functions of (P)RR in this study. In the representative IHC photos, brown color indicates (P)RR protein staining. Villin: a protein that mainly localizes in gut epithelium. TdTomato: a red fluorescent protein with an excitation peak of 554nm and an emission peak of 581nm *in vitro*.

**
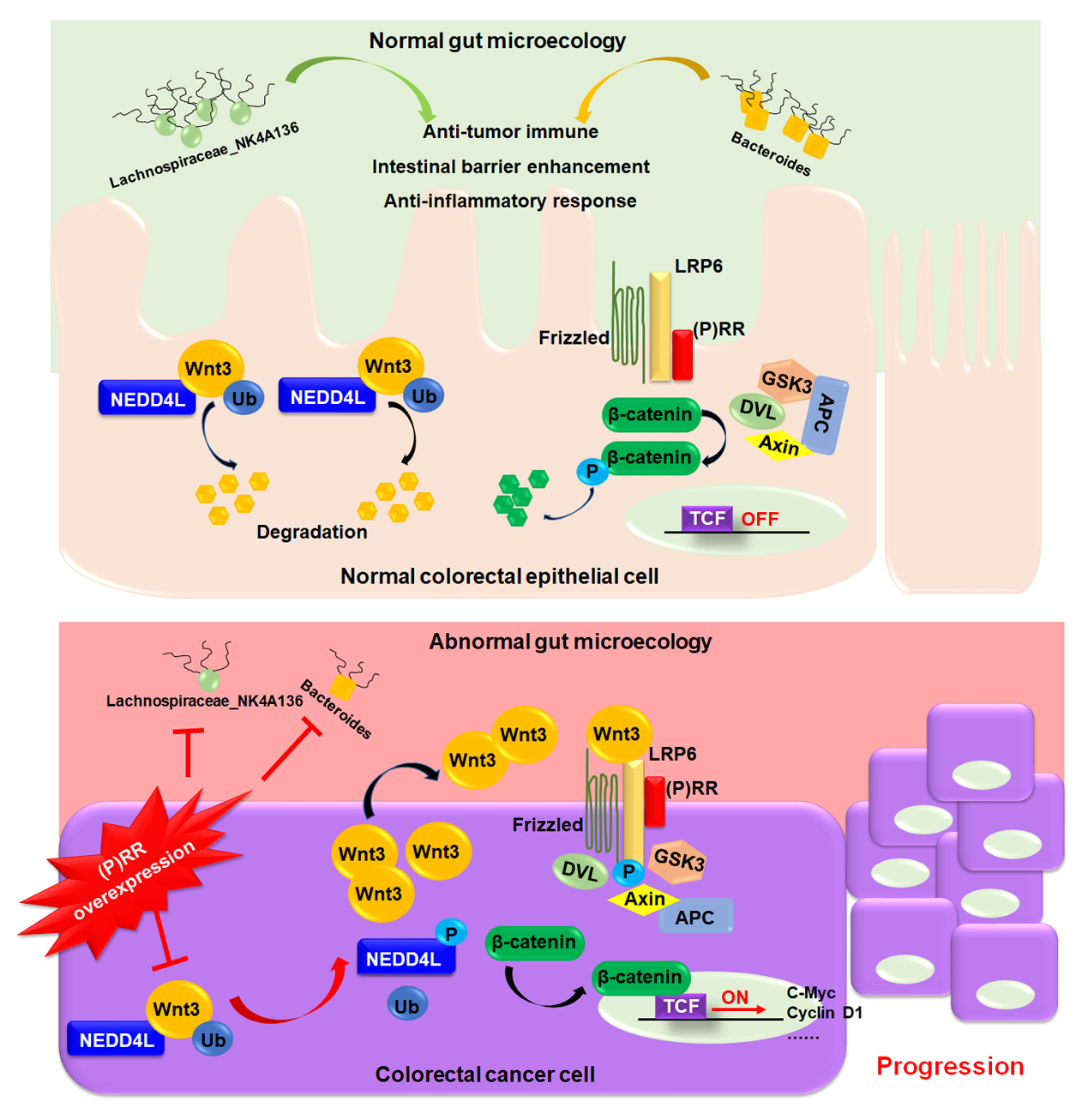
**

**Supplementary figure 3.** (P)RR promotes CRC progression through inhibiting the NEDD4L-mediated Wnt3 ubiquitination and modulating gut microbiota. In normal colorectal epithelial cells, the E3 ubiquitin ligase NEDD4L mediates Wnt3 ubiquitination, thus triggers subsequent Wnt3 degradation. Without excessive Wnt3, Wnt/β-catenin signaling is maintained at a normal level. Additionally, some probiotics, especially Lachnospiraceae_NK4A136 and Bacteroides genus, also help maintain the normal gut microecology and protect normal cells against cancerization. Probiotics exert the protective effects by contributing to the anti-cancer immune, intestinal barrier enhancement and anti-inflammatory response. In CRC cells, aberrant (P)RR overexpression inhibits the NEDD4L-mediated Wnt3 ubiquitination. So that Wnt3 could escape from the ubiquitination-triggered degradation. As a result, accumulating Wnt3 will bind to Wnt receptor complex, thus excessively activate Wnt signaling activity. Consequently, Wnt target oncogenes as c-Myc and Cyclin D1 will aberrantly express. In addition, (P)RR overexpression also leads to abnormal gut microecology, especially, Lachnospiraceae_NK4A136 and Bacteroides genus that are generally protective against CRC are suppressed. Finally, aberrant (P)RR overexpression leads to CRC progression.
